# Supplementary material for: Dual-Responsive Starch Hydrogels via Physicochemical Crosslinking for Wearable Pressure and Ultra-Sensitive Humidity Sensing
Source: Sensors (Basel). 2025 Aug 13;25(16):5006. doi: 10.3390/s25165006 (PMC12390084; doi:10.3390/s25165006)
Supplement: Supplementary file 1 [file sensors-25-05006-s001.zip › Supporting Information.pdf]

Supporting information for

Dual-Responsive Starch Hydrogels via Physicochemical Crosslinking for Wearable Pressure and Ultra-sensitive Humidity Sensing

Zi Li[1,2], Jinhui Zhu[1,2], Zixuan Wang[1,2], Hao Hu[1,2,*], Tian Zhang[1,2,*]

[1] Electronic Information School, Wuhan University, Wuhan 430072, P.R. China

[2] Suzhou institute of Wuhan University, Suzhou215000, P.R. China

Corresponding authors: Hao Hu 2023102120043@whu.edu.cn and Tian Zhang txz908@whu.edu.cn

The Following Supplementary documents are contained below:

Figures S1-S9.

Supplementary Movie S1: Force sensitivity of SPG hydrogel.

Supplementary Movie S2: Humidity sensitivity of D-SPG film.

Figures

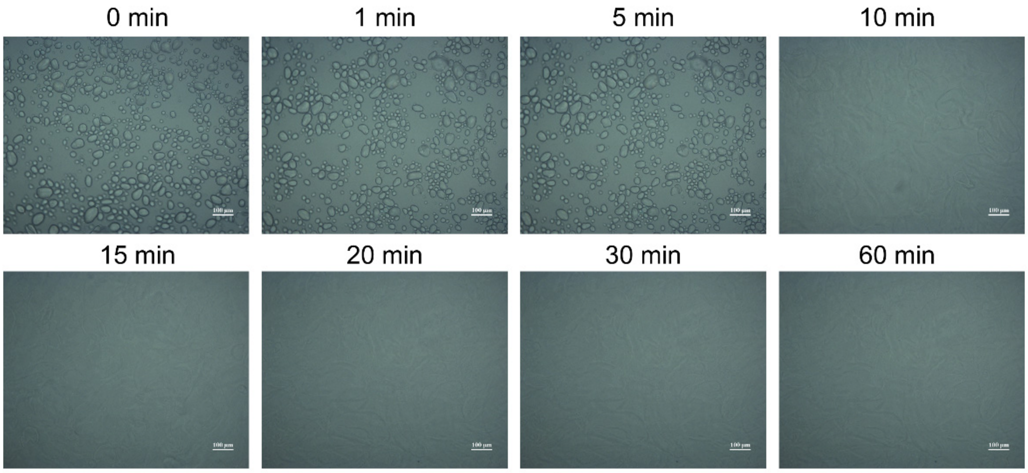

Figure S1. Starch gelatinization under 80 °C water bath.

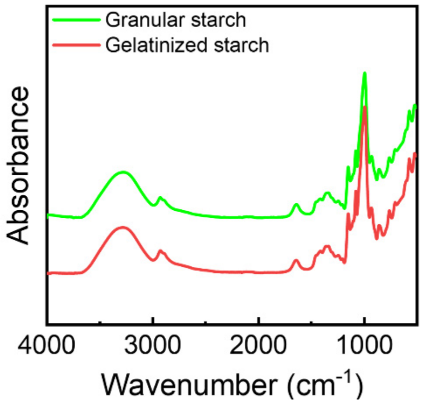

Figure S2. The FTIR of granular and gelatinized starches.

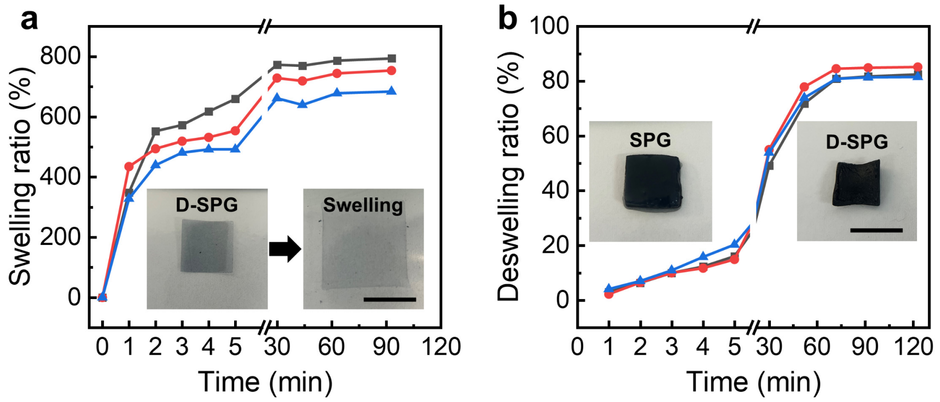

Figure S3. The swelling curve of D-SPG (a) and the deswelling curve of SPG (b). The inserted photographs show the initial and stable states after swell/deswelling treatments. The swelling condition of D-SPG is room temperature deionized water. The deswelling condition of SPG is a 50℃ oven. The scale bars are 1 cm.

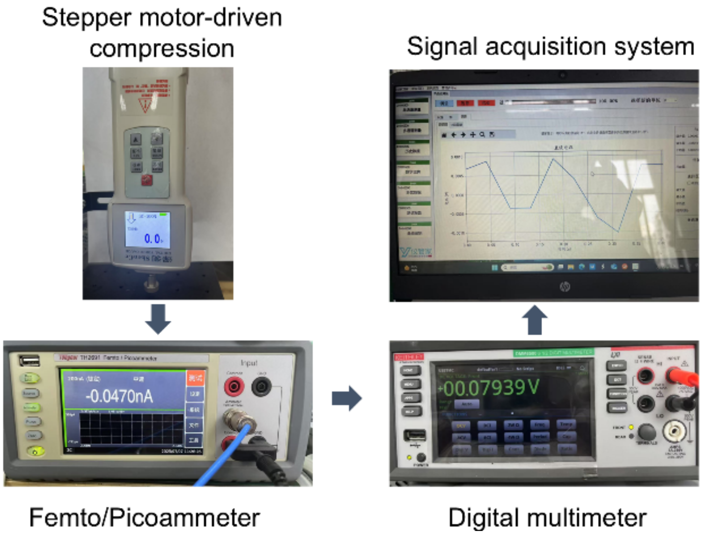

Figure S4. The photographs of electromechanical coupling system.

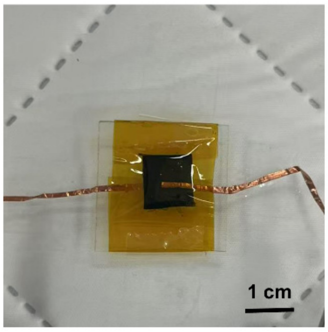

Figure S5. The photographs of SPG hydrogel as a force-electric sensor.

.

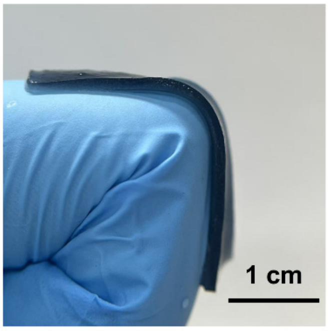

Figure S6. The photographs of SPG hydrogel applied on finger joints.

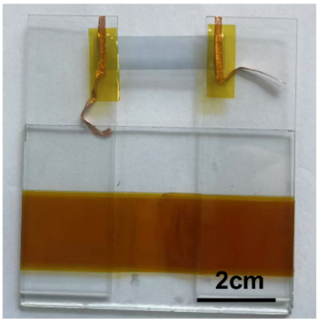

Figure S7. The photographs of D-SPG film as a humidity-electric sensor.

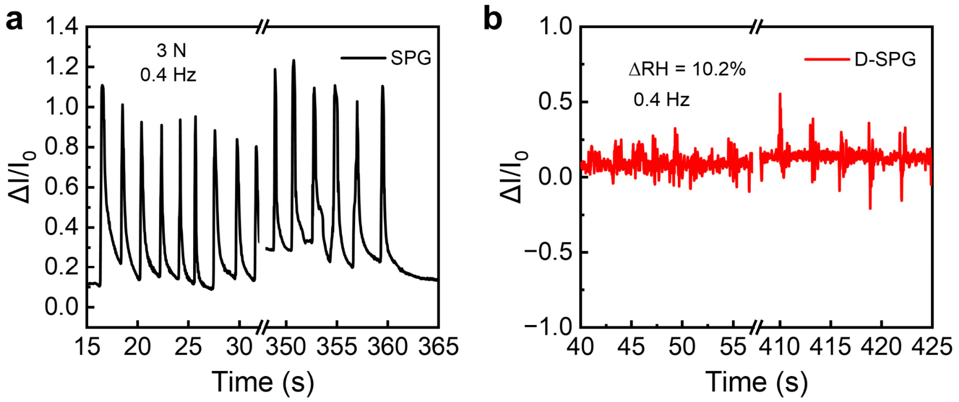

Figure S8. Cycle stability testing of SPG (a) and D-SPG (b)

	Stimulus	Sensitivity	Response Time	Signal Stability	Mechanical Strength	Drifit	Conductivity
SPG	Pressure	-----	0.15 s	> 200	266 kPa	2.7%	10^{-7} S/cm
D-SPG	Humiidity	ΔRH=6.6%	0.12 s	> 200	-----	----	10^{-8} S/cm

Figure S9. Summary tables of SPG and D-SPG.
